# Developmental defects of enamel in primary teeth - findings of a regional German birth cohort study

**DOI:** 10.1186/s12903-016-0235-7

**Published:** 2016-07-07

**Authors:** Yvonne Wagner

**Affiliations:** Department of Preventive Dentistry and Pediatric Dentistry, Jena University Hospital, Bachstr. 18, Jena, Germany

**Keywords:** Enamel defects, Prevalence, Primary dentition, Enamel hypomineralisation

## Abstract

**Background:**

The aim was to assess the prevalence, distribution and associated risk factors of developmental defects of enamel (DDE) in 3-year-old Thuringian children in 2013 as part of a prospective cohort study.

**Methods:**

The subjects (*n* = 377) were all participants in a Thuringian oral health programme. Children of the birth cohort 2009/2010 were invited to dental examination in the first year of life, followed up with continuous dental care over the next 3 years. Dental caries was scored using the WHO diagnostic criteria expanded to the d1-level without radiography. Enamel defects were assessed according to the modified DDE Index. Data were analysed statistically (multivariate logistic regression).

**Results:**

The children were aged 3.3 ± 0.7 years and 52.5 % of them were male. Caries prevalence was 15.6 % and caries experience 0.9 ± 3.3 d_1-4_mfs. The prevalence of DDE was 5.3 % with an average of 2.7 (±1.4) affected teeth. Second primary molars were the most affected teeth and demarcated opacities the most prevalent type. No child had Amelogenesis imperfecta and six children showed hypomineralised second primary molars. Enamel defects were associated with preterm birth (*p* = 0.024; OR = 4.9) and hospitalisation in the first year of life (*p* = 0.013; OR = 4.6).

**Conclusion:**

A relatively small proportion of 3-year-old Thuringian children suffered from DDE, with second primary molars as the most affected teeth and demarcated opacities as the most prevalent type of defect. Preterm birth and hospitalisation in first year of life can be considered as risk factors for DDE in the primary dentition.

**Trial Registration:**

German Clinical Trials Register, DRKS00003438

## Background

Recently there has been an increase in awareness of the role of non-fluoride-associated developmental defects of enamel (DDE) in the primary dentition [1–3]. DDE can have a significant impact on oral health and aesthetic appearance; children with DDE may suffer from tooth sensitivity, increased caries susceptibility and altered occlusal function [1–4]. Additionally, developmental disturbances of the enamel in the primary dentition may be predictive of similar alterations in the permanent dentition [5–8]. DDE are variations in quality and quantity of the enamel, resulting from disturbances in the amelogenesis process [9, 10]. Enamel hypomineralisation is a qualitative defect presenting alterations in enamel translucency and opacity. The defective enamel is of normal thickness, and opacities can be diffuse or demarcated with white, yellow or brown colour [9, 10]. Enamel hypoplasia is a quantitative enamel deficiency and presents a decreased enamel thickness such as pits, grooves or generalised lack of surface enamel [9, 10]. Much of this demarcated qualitative DDE would currently be described as molar-incisor hypomineralisation (MIH) in the permanent dentition or hypomineralised second primary molar (HSPM) in the primary dentition [8, 11–14]. The prevalence of DDE in the primary dentition varies between 4 and 75 %, depending on the population studied and the criteria used for scoring [7, 15–20]. In recent years, published studies have increasingly focused on the prevalence and aetiology of DDE, particularly on the determination of DDE in the permanent dentition [1, 21]. However, an increase in the prevalence of DDE has not been proved [11, 22]. Several clinical indices have been developed to categorise enamel defects (for example the Dean-Index, the DDE Index, the modified DDE Index (mDDE) and the evaluation criteria of the EAPD), with the result that the conducted studies are not comparable [9, 11, 23–25]. The EAPD criteria are based on the mDDE and have advantages with respect to hypomineralised lesions owing to the scoring of post-eruptive enamel loss, atypical caries, atypical restoration and atypical extraction [11].

Currently, the aetiology of DDE is still not completely clear and the causes are controversial [14–16, 21]. Development of the primary teeth starts during pregnancy and the amelogenesis completes around 12 months after birth [10]. During this tooth development time, a series of factors can interact, accumulate or combine to affect the ameloblasts, disrupt matrix formation or maturation and lead to DDE [10]. Several factors have been suggested as associated with the development of the defect, such as pre-, peri- and postnatal problems and local, systemic or genetic conditions [7, 14–17, 21]. They range from maternal factors, such as age at the birth of the child, social influences, diseases or infections during pregnancy (pre-eclampsia, diabetes, rubella), malnutrition, use of anti-allergic medicines or anti-asthmatic medicines, alcohol consumption or smoking during the prenatal period, dioxins or Bisphenol A exposure, and prematurity to various child factors, including low birth weight, Apgar score, fever, infectious and other diseases, lack of breastfeeding or prolonged breastfeeding, nutritional problems, use of the antibiotic amoxicillin, hyperbilirubinemia, and respiratory distress, among others [4, 7, 14–17, 21, 25–41].

The purpose of this study was to assess the prevalence, distribution and associated risk factors of DDE in 3-year-old Thuringian children as part of a prospective cohort study. Although, the topic has already been explored, there are no studies in this population and in this age group with a longitudinal data collection [1, 11–20]. Studies in the primary dentition are limited and most of them have a cross-sectional design or are retrospective [1, 11–20]. The examination of a younger age group and following them over time allows a more accurate assessment of possible influencing early life factors and their relationship with DDE.

## Methods

### Study design

This study was part of a prospective cohort study to evaluate the impact of a preventive programme on the oral health of Thuringian children in Germany (German Clinical Trials Register DRKS00003438). The Ethics Committee of Jena University Hospital approved the study (registration number 2759-02/10). The study was conducted in full accordance with the ethical requirements of the World Medical Association Declaration of Helsinki (2008).

### Study population

The study was conducted in a medium-sized and well-situated city in Germany (Jena, Thuringia) with a relatively low proportion of families with a low socio-economic status (SES) or migration background. The children of the Jena birth cohort 7/2009 to 10/2010 (*n* = 1162) were invited to have a dental examination in the first year of life. Those families who accepted the invitation (*n* = 512) were included as participants of the preventive programme with caries-risk-related continuous dental care from birth up to the age of 3 years. A caries risk assessment was carried out to categorise the children using the Caries-risk Assessment Tool for infants, children and adolescents of the American Academy of Pediatric Dentistry (AAPD) [42]. Children with an increased caries risk were reappointed every three months and children with a low or moderate caries risk every six months. High-risk children received fluoride varnish application biannually. A thin film of fluoride varnish was applied by a dentist in the dental practice, using <0.25 ml per child, limited to those surfaces at risk. The study population included all the children who had participated in the final examination of the preventive programme (*n* = 377, 32.4 %). The eligibility criteria were provision of written consent by the caregiver and availability of data relating to the caregiver’s interview and dental examination of the child. The exclusion criteria were no written consent and incomplete data.

### Dental examination

The children were examined at the Department of Preventive and Paediatric Dentistry, Jena University Hospital, Germany. The examinations were conducted using a dental light, mirror and sterile gauze for teeth cleaning and drying. Dental caries was scored using the WHO diagnostic criteria expanded to the d1-level without radiography [43]. Enamel defects were assessed according to the mDDE [9]. The scoring was at surface level, with three surfaces (buccal/labial, lingual/palatal and occlusal/incisal) for each tooth. An enamel defect of ≤1 square millimetre was considered as sound [22]. The visual clinical presentation of the DDE was assessed according to these types:

1. Demarcated opacity-a qualitative defect presenting alterations in enamel translucency and opacity. The defective enamel is of normal thickness and is white, creamy, yellow or brown in colour. There is a clear border with adjacent normal enamel [9]. Demarcated opacities are relatively prone to caries and enamel substance loss (post-eruptive enamel breakdown) [4, 11, 12]. This category also includes HSPM [11, 12]. A child was classified to have HSPM when at least one second primary molar was diagnosed with HSPM [11].
2. Diffuse opacity-a qualitative defect presenting alterations in enamel translucency and opacity. The defective enamel is of normal thickness and is white in colour. It can have a linear, patchy or confluent distribution, and there is no clear border with adjacent normal enamel. It also includes opacities owing to fluorosis [9].
3. Hypoplasia-a quantitative enamel deficiency with a decreased enamel thickness such as pits, grooves or larger areas of missing enamel [9].
4. Combination of defects (demarcated and diffuse opacities, hypoplasia and opacities) [9].
5. Amelogenesis imperfecta (AI)-a range of genetically caused enamel malformations (hypoplasia, hypocalcification and hypomaturation) [44].

The size of the defect (lesion extension criteria) was recorded in thirds of the affected tooth surface area: less than one-third of the tooth surface affected, at least one-third but less than two-thirds of the surface area affected, and at least two-thirds of the tooth surface affected [9, 11].

All records were performed by the same calibrated clinician (YW), who had been trained and calibrated following WHO guidelines by an experienced epidemiologist [43]. The training was performed using a set of photographs with different clinical situations (teeth with enamel defects, including MIH, HSPM, carious lesions, dental fluorosis, AI). A training exercise was then carried out, involving the clinical oral examination of 10 children in the same age group as those of the main study. Afterwards, the calibration was performed. The dentist examined a group of 25 pre-selected subjects twice on successive days to assess the consistency. The intrarater-reliability regarding the DDE prevalence was very good (*k* = 0.83).

During the main survey duplicate examinations at the beginning, after 1 year and at the end of the survey were conducted with 25 subjects each randomly selected. The intrarater-reliability regarding the DDE prevalence was very good and ranged from 0.81 to 0.84.

### Questionnaire

Furthermore, a standardised questionnaire was conducted, which was updated during each dental visit. The questionnaire collected the following information: age, gender, migration background, special health care needs, diseases during pregnancy, type of delivery (Caesarean, vaginal), preterm birth (<37 weeks of pregnancy), weight at birth (<2.500 grams), general diseases (cardiovascular, metabolic or kidney disease), hospitalisation in the first year of life, systemic infectious diseases (pneumonia, otitis media, viral gastroenteritis, chickenpox, etc.), breathing patterns, allergies, medication, systemic antibiotic medication, feeding behaviour, the use of vitamin D or fluoride supplements, oral hygiene, and the SES of the families. The SES was recorded using the Brandenburg social index [45]. The index was computed for each child based on the education and employment status of the parents, and children were allocated to lower, middle or higher SES groups. For cases with missing values of one parent, the value of the other parent was double weighted, analogous for single parents [45].

The development of the questionnaire and the selection of items were based on the assumption that early childhood caries and DDE could share possible aetiological factors [36, 46]. The developed questionnaire was tested regarding face validity and content validity using a panel of experts (dentists of the Department of Preventive and Paediatric Dentistry, Jena University Hospital, Germany) and respondents (randomly selected parents attending the Department of Preventive and Paediatric Dentistry, Jena University Hospital, Germany for routine dental examinations). The revised questionnaire was then tested in a pilot test by collecting data from 25 randomly selected parents not included in the final sample.

### Statistical analysis

Data were recorded in Microsoft Excel files (Office Version 2011, Microsoft Corporation, Redmond, WA, USA) and transferred to Statistical Package for Social Sciences (SPSS version 20) for analysis (IBM Corporation, Armonk, NY, USA). After correlation analysis (Pearson) and adjustment of variables that showed a strong or very strong correlation (correlation coefficient >0.5), the chi-square test (Pearson) or Fisher exact test was used to determine the statistical significant associations between the independent variables (low SES, ethnicity, diet, preterm birth, general disease, medication, use of vitamin D supplements, etc.) and the outcome variable enamel defects before the multivariate analysis was conducted. Variables that showed significant associations (*p* < 0.2) were included in the multivariate logistic regression analysis. A backward stepwise elimination was used in the logistic regression. A further calculation was conducted to determine whether there was a difference between the independent variables (gender, migration background, low SES, diseases during pregnancy, Caesarean type of delivery, preterm birth, low birth weight) of the participating children and the children who dropped out. The data were analysed using the *t*-test. A p-value ≤0.05 was set to indicate statistically significant differences.

## Results

A total of 377 children (mean age 3.3 ± 0.7 years; 52.5 % male; dropout 26.4 %) were examined. Major causes of dropout were schedule difficulties (*n* = 67), relocation to another area (*n* = 12) and 56 children dropped out without stating reasons. There were no differences, with the exception of low SES, between participating children and children who were lost owing to dropout regarding their gender, migration background, diseases during pregnancy, Caesarean type of delivery, preterm birth and low birth weight (Table 1). Table 2 presents the results of the final dental examination. Caries prevalence (d1-level) was 15.6 % (*n* = 59) and caries experience 0.9 ± 3.3 d_1-4_mfs. The prevalence of DDE was 5.3 % (*n* = 20) at the child level. The mean number (±SD) of DDE teeth per child of the children who had defects was 2.7 (±1.4). From the first dental visit to the final examination in six children the DDE was no longer detectable owing to tooth wear. The data of DDE distribution according to tooth type for the affected children are shown in Table 3. The majority of children with DDE had demarcated opacities (75.0 %); 15.0 % of the children had hypoplastic defects; and diffuse opacities were the least common ones (5.0 %). No child had AI, and six (30.0 %) children showed HSPM. Second primary molars (35.2 %), canines (30.8 %) and incisors (29.7 %) were more affected than first primary molars (4.4 %) (*p* < 0.001). The distribution of affected teeth between maxilla (51.6 %) and mandible (48.4 %) was close to even. Most DDE (90.0 %) extended across less than one-third of the surface area of the affected teeth. All DDE on the tooth surface were located on the buccal and occlusal areas. Descriptions of the independent variables of all children and of the children with enamel defects are presented in Table 4. Statistically significant associations were found between enamel defects in children with preterm birth and low birth weight (OR = 4.67), children with general disease and special health care needs (OR = 2.45), children with hospitalisation in the first year of life (without preterm birth/low birth weight children) (OR = 4.44), and children with systemic antibiotic medication (OR = 2.21). The association between DDE and caries was not statistically significant (*p* = 0.538) (Table 4). The results of the final multivariate logistic regression analysis (Table 5) demonstrate associations between DDE in children and preterm birth and low birth weight, general disease and special health care needs, hospitalisation in the first year of life (without preterm birth and low birth weight children) and systemic antibiotic medication. Children with preterm birth and low birth weight had a 4.9 times higher probability of having DDE in their primary teeth than children with full-term birth and normal birth weight. Children with hospitalisation in the first year of life (without preterm birth and low birth weight children) had a 4.6 times higher probability of having DDE than children with no hospitalisation.

**Table 1:** Description of independent variables for children in preventive programme and children who were lost due to dropout

Variables	All children (*n* = 512)		Children in preventive programme (*n* = 377)		Children who dropped out (*n* = 135)		p-value
	%	n	%	n	%	n	
Gender male	52.0	270	52.3	197	54.1	73	0.763
Migration background	6.3	32	6.1	23	6.7	9	0.837
Low socioeconomic status	13.2	68	9.3	35	24.4	33	0.001
Diseases during pregnancy	31.6	162	34.2	129	32.5	44	0.752
Caesarean type of delivery	24.6	126	27.1	102	25.3	34	0.822
Preterm birth/low birth weight	4.3	22	4.2	16	4.4	6	1.000

*p* < 0.05, statistically significant

**Table 2:** Caries prevalence, caries experience and developmental defects of enamel (DDE) in children who participated in the final dental examination

		Final examination (*n* = 377)
Age	Mean ± SD	3.3 ± 0.5 years
Male	N (%)	197 (52.3)
Low socioeconomic status	N (%)	35 (9.3)
Caries prevalence (d1-4)	N (%)	59 (15.6)
d_1-4_mfs	Mean ± SD	0.9 ± 3.3
DDE prevalence	N (%)	20 (5.3)
Number of DDE teeth per child of the children who had defects	Mean ± SD	2.7 ± 1.4

**Table 3:** Distribution of types and size of developmental defects of enamel according to tooth type for affected children (*n* = 20)

Type of defect	Children (*n* = 20)	Tooth									
		55/65	54/64	53/63	52/62	51/61	71/81	72/82	73/83	74/84	75/85
Demarcated opacities % (n)	75.0 (15)										
White	35.0 (7)	15.0 (3)	5.0 (1)	5.0 (1)	5.0 (1)	10.0 (2)	10.0 (2)	0.0 (0)	10.0 (2)	0.0 (0)	15.0 (3)
Yellow	40.0 (8)	15.0 (3)	5.0 (1)	10.0 (2)	0.0 (0)	5.0 (1)	10.0 (2)	0.0 (0)	15.0 (3)	5.0 (1)	15.0 (3)
Brown	0.0 (0)	0.0 (0)	0.0 (0)	0.0 (0)	0.0 (0)	0.0 (0)	0.0 (0)	0.0 (0)	0.0 (0)	0.0 (0)	0.0 (0)
Hypomineralised second primary molar	30.0 (6)	30.0 (6)	-	-	-	-	-	-	-	-	30.0 (6)
Diffuse opacities % (n)	5.0 (1)										
Lines	0.0 (0)	0.0 (0)	0.0 (0)	0.0 (0)	0.0 (0)	0.0 (0)	0.0 (0)	0.0 (0)	0.0 (0)	0.0 (0)	0.0 (0)
Patchy	5.0 (1)	5.0 (1)	0.0 (0)	5.0 (1)	0.0 (0)	0.0 (0)	0.0 (0)	0.0 (0)	5.0 (1)	0.0 (0)	5.0 (1)
Hypoplasia % (n)	15.0 (3)										
Pits	15.0 (3)	5.0 (1)	0.0 (0)	10.0 (2)	0.0 (0)	10.0 (2)	15.0 (3)	0.0 (0)	0.0 (0)	0.0 (0)	0.0 (0)
Grooves	0.0 (0)	0.0 (0)	0.0 (0)	0.0 (0)	0.0 (0)	0.0 (0)	0.0 (0)	0.0 (0)	0.0 (0)	0.0 (0)	0.0 (0)
Larger areas	0.0 (0)	0.0 (0)	0.0 (0)	0.0 (0)	0.0 (0)	0.0 (0)	0.0 (0)	0.0 (0)	0.0 (0)	0.0 (0)	0.0 (0)
Combination % (n)	5.0 (1)	5.0 (1)	0.0 (0)	0.0 (0)	0.0 (0)	0.0 (0)	0.0 (0)	0.0 (0)	0.0 (0)	0.0 (0)	5.0 (1)
Size											
$<\frac{1}{3}$ tooth surface	90.0 (18)	35.0 (7)	10.0 (2)	30.0 (6)	5.0 (1)	25.0 (5)	35.0 (7)	0.0 (0)	30.0 (6)	5.0 (1)	35.0 (7)
$\frac{1}{3}-\frac{2}{3}$ tooth surface	10.0 (2)	10.0 (2)	0.0 (0)	0.0 (0)	0.0 (0)	0.0 (0)	0.0 (0)	0.0 (0)	0.0 (0)	0.0 (0)	5.0 (1)
$>\frac{2}{3}$ tooth surface	0.0 (0)	0.0 (0)	0.0 (0)	0.0 (0)	0.0 (0)	0.0 (0)	0.0 (0)	0.0 (0)	0.0 (0)	0.0 (0)	0.0 (0)

**Table 4:** Description of independent variables for children in preventive programme and children with enamel defects. Chi-square test or Fisher exact test were used with enamel defects as the dependent variable

Variables	Children in preventive programme (*n* = 377)			Children with enamel defects (*n* = 20)				
		%	n	%	n	UnadjustedOR	95 % CI	p-value
Gender	Male	52.3	197	6.1	12	1.37	0.55–3.44	0.647
	Female	47.7	180	4.5	8			
Migration background	Yes	6.1	23	5.0	1	0.94	0.12–7.37	1.000
	No	93.9	354	5.3	19			
Low socioeconomic status	Yes	9.3	35	3.1	1	0.55	0.07–4.28	0.714
	No	90.7	342	5.5	19			
Diseases during pregnancy	Yes	34.2	129	4.7	6	0.82	0.31–2.17	0.811
	No	65.8	248	5.6	14			
Caesarean type of delivery	Yes	27.1	102	4.9	5	0.89	0.31–2.52	1.000
	No	72.9	275	5.5	15			
Preterm birth/low birth weight	Yes	4.2	16	20.0	3	4.67	1.21–17.95	0.046
	No	95.8	361	4.7	17			
General disease/special health care needs	Yes	7.2	27	13.0	3	2.45	0.67–8.94	0.165
	No	92.8	350	4.8	17			
Hospitalisation first year of life	Yes	10.3	39	17.9	7	5.47	2.03–14.69	0.002
	No	89.7	338	3.8	13			
Hospitalisation first year of life without premature/low birth weight children^a^	Yes	6.1	23	17.4	4	4.44	1.35–14.60	0.027
	No	93.9	354	4.5	16			
Systemic infectious disease	Yes	69.0	260	6.2	16	1.85	0.06–5.67	0.329
	No	31.0	117	3.4	4			
Mouthbreathing	Yes	0.8	3	0.0	0	Mathematically incalculable		1.000
	No	99.2	374	5.3	20			
Allergies	Yes	0.8	3	0.0	0	Mathematically incalculable		1.000
	No	99.2	374	5.3	20			
Breastfeeding	Yes	67.4	254	4.7	12	0.71	0.28–1.79	0.625
	No	32.6	123	6.5	8			
Medication	Yes	13.5	51	8.9	4	1.65	0.53–5.14	0.329
	No	86.5	326	4.8	16			
Systemic antibiotic medication	Yes	20.4	77	9.1	7	2.21	0.85–5.74	0.148
	No	79.6	300	4.3	13			
Use of vitamin D supplements	Yes	95.8	361	5.2	19	1.20	0.15–9.57	0.590
	No	4.2	16	6.3	1			
Use of fluoride supplements	Yes	15.9	60	5.0	3	0.93	0.26–3.27	1.000
	No	84.1	317	5.4	17			
Caries	Yes	15.6	59	6.8	4	1.37	0.44–4.26	0.533
	No	84.4	318	5.0	16			

*p* < 0.2, statistically significant

^a^Preterm birth/low birth weight and hospitalisation, correlation coefficient (Pearson) = 0.691

**Table 5:** Multivariate logistic regression analysis of associations between enamel defects in children and preterm birth and low birth weight, general disease and special health care needs, hospitalisation in the first year of life (without preterm birth and low birth weight children), and systemic antibiotic medication. Backward stepwise elimination was used

Variable		OR	95 % CI	p-value
Preterm birth/low birth weight	Yes	4.9	1.23–19.26	0.024
	No (ref)			
Hospitalisation first year of life without preterm birth/low birth weight children	Yes	4.6	1.37–15.40	0.013
	No (ref)			

## Discussion

The present study is based on data from a regional German birth cohort study. Clinical and survey data were obtained on an ongoing basis and at regular intervals up to the age of 3 years. It was determined that 5 % of the 3-year-olds had at least one tooth with a defect of the enamel in their primary dentition and that second primary molars were the most affected teeth and demarcated opacities the most prevalent type. The results of this study are consistent with previous studies concerning the prevalence of enamel defects in the primary dentition [5, 19]. A birth-cohort study of healthy, well-nourished children (698 4- to 5-year-olds) in Iowa found that 6 % of the children examined had enamel hypoplasia and 27 % isolated opacities [19]. A study among Mexican children showed that 10 % exhibited DDE [5].

In this study, the distribution of DDE revealed that second primary molars, canines and incisors were most frequently affected. These findings coincide with a study among Brazilian children [20]. The relatively high prevalence of DDE in second primary molars is a common observation [8, 11–13, 16, 29]. Studies suggest a relationship between the presence of HSPM in the primary dentition and the development of MIH in the permanent dentition [6, 8, 11–13, 16, 22]. Development of the second primary molar and the first permanent molar starts at the same time, so that occurring risk factors could influence both dentitions [10]. However, the underlying causative mechanism is inconclusive and a genetic and environmental influence is assumed [6, 8, 29]. Several factors have been identified as determinants for MIH, whereas no association with HSPM was found [6, 28]. It can be expected that there are several commonly occurring factors for the development of HSPM that are more pre- and perinatal than postnatal [10, 29].

The finding that demarcated opacities were the most prevalent type agrees with those of Clarkson and O’Mullane [23], Correa-Faria et al. [26] and Cruvinel et al. [7], but disagrees with Lunardelli and Peres [20] and Masumo et al. [36], who found that diffuse opacities were more prevalent [7, 20, 23, 26, 36].

In the present study the dentist was able to identify DDE before post-eruptive enamel breakdown or the onset of caries could occur owing to the prospective study design from birth to early childhood. By comparison, most studies are retrospective or have a cross-sectional design, presenting biased data owing to the limited memory of the parents and missing longitudinal examination data [1, 11–20]. Moreover, teeth with a carious lesion are often excluded from the analysis or recorded as decayed so that the underlying enamel defect remains masked and undiagnosed, and the correlation between DDE and caries may be underestimated [15, 21]. Teeth with DDE are more susceptible to caries [2–4]. Children with enamel hypoplasia seem to be especially at high risk for caries and coined the term hypoplasia-associated early childhood caries [3]. Less mineralisation, porosity and irregular surfaces of teeth with DDE allow plaque accumulation and limit oral hygiene [2–4]. Despite the preventive programme four children with DDE developed a carious lesion. Although, the observed association between DDE and caries in the present study was not statistically significant, it can be assumed that DDE are a risk factor for the development of a carious lesion. Parents need to be aware of caries and DDE. Early maternal counselling and continuous risk-orientated dental care may be an approach for preventing dental caries, and to promote awareness of the importance of a healthy diet and good oral hygiene [47]. Unfortunately, the existing evidence and efficiency of any specific clinical, behavioural or community-based intervention or programme for caries prevention remain limited [47].

Another interesting finding of the present study was that in six children the DDE was no longer detectable at the age of 3 years owing to tooth wear. Teeth with DDE are predisposed to tooth wear (attrition, abrasion, erosion) owing to the enamel being thinner or hypomineralised [3, 5, 15]. This result confirms the strength of the present study and suggests that the determined DDE prevalence in cross-sectional studies may be underestimated.

The present study showed that children with a history of preterm birth and low birth weight have a significantly higher prevalence of DDE. These findings were confirmed by several other studies showing that preterm birth is a predisposing factor for DDE [4, 7, 27, 31–37]. Preterm birth is often associated with low birth weight and neonatal complications such as anaemia, calcium deficiency, infections, respiratory diseases, orotracheal intubation and ventilation [4, 7, 27, 31–37]. Oxygen deprivation may alter ameloblastic cell function [7]. In addition, preterm-born children are usually in need of special health care, hospitalisation and medication, including systemic antibiotic medication [7]. These factors can interact, accumulate or combine to raise disease risk, and it is difficult to distinguish them [21, 31–36]. The observed association between preterm birth, low birth weight and hospitalisation in the first year of life and DDE in this study suggests the influence of these factors on the development of the primary dentition. Children with hospitalisation in the first year of life showed an increased risk of DDE. Even though other factors showed no statistically significant association with DDE in the multivariate analysis, a trend was observable. It can be assumed that the occurrence of several possible factors such as preterm birth, general diseases, systemic antibiotic medication and hospitalisation could influence tooth development.

The study did have a few limitations. First, this study was limited to a relatively small geographic location and to those children who participated in the preventive programme. Consequently, the findings are restricted to this population group. Owing to the relatively small sample size, the high dropout rate and the lack of randomisation, the prevalence of DDE could be over- or underestimated. To reduce the source of potential bias, the characteristics (gender, low SES, migration background, diseases during pregnancy, type of delivery, preterm birth and low birth weight) of participating children and children who were lost owing to dropout were compared. A statistically significant difference was found for the variable low SES (Table 1). Families with a low SES were more likely to drop out [48]. Lower-income groups usually have a lower response rate to health promotion and preventive programmes [48]. The present study found no association between the low SES factor and DDE. However, a relationship between social factors and DDE has been suggested owing to underlying risk behaviours, inadequate nutrition and less pregnancy care [3, 37]. Therefore, a comparison with national and international studies on DDE in the primary dentition should be made with caution owing to the limited available data, variations in study design, the diagnostic criteria and the socio-behavioural, environmental and genetic differences of the population studied.

The present study demonstrated that a relatively small proportion of 3-year-old Thuringian children suffered from DDE and that the second primary molars were the most affected teeth with demarcated opacities as the most prevalent type of defect. Additionally, the study found an association between enamel defects and preterm birth, low birth weight and hospitalisation in the first year of life.

## Conclusion

A relatively small proportion of 3-year-old Thuringian children suffered from DDE, with second primary molars as the most affected teeth and demarcated opacities as the most prevalent type of defect. Preterm birth, and hospitalisation in first year of life can be considered as risk factors for DDE in the primary dentition.

## Abbreviations

AAPD, American Academy of Pediatric Dentistry; AI, Amelogenesis imperfecta; DDE, developmental defects of enamel; HSPM, hypomineralised second primary molar; SES, socio-economic status
